# RETROFIT-LAT: A comprehensive dataset for energy efficiency investments in Latvia

**DOI:** 10.12688/openreseurope.20042.2

**Published:** 2025-07-31

**Authors:** Αlexandros Menelaos Tzortzis, Panagiota Rempi, Sotiris Pelekis, Aija Zucika, Christos Ntanos, Askounis Dimitris

**Affiliations:** 1 Decision Support Systems Lab, National Technical University of Athens, Zografou, Attica, Greece; 2 VIDES INVESTICIJU FONDS SIA, Riga, Latvia

**Keywords:** Energy efficiency, Renovation measures, Investment financing, Energy emission reduction, Renewable energy sources

## Abstract

This article presents RETROFIT-LAT: a collection of data from 1010 residential building projects, funded by the Republic of Latvia’s Environmental Investment Fund (LEIF). The first dataset analyses the energy performance and sustainability of buildings before and after retrofitting actions, including their energy consumption, *CO*_2_ emissions, and energy classes. It spans projects implemented from 1870 to 2022, covering various building types and regions, with data on their energy use, heat loss coefficients, and both renewable and non-renewable energy contributions. The second dataset focuses on photovoltaic (solar panel) installations as part of energy efficiency measures. It documents electricity consumption, primary energy use before and after installation, inverter power, and reductions in *CO*_2_ emissions. By making this data available in a structured and analyzable format, RETROFIT-LAT aims to support the development of data-driven assessments, including machine learning models for forecasting retrofit outcomes. It is intended to assist researchers, engineers, and policymakers in evaluating and designing effective energy efficiency strategies for residential buildings.

## Specifications table

**Table:** 

**Subject**	Environmental Engineering, Artificial Intelligence
**Specific Subject Area**	Energy consumption and CO _2_ emission of buildings, energy efficiency activities, building data, project costs and grants data
**Type of Data**	Data in comma seperated values format (.csv) Python (.py) scripts used for preprocessing
**Data format**	Pre-processed/pseudo-anonymized data in pivot tables
**Data collection**	RETROFIT-LAT comprises information on projects that received co-financing for renovations. Prior to renovation, details on energy consumption, building characteristics, and investment costs were provided by project applicants. Post-renovation data was gathered directly from building stakeholders. Data were collected from all completed projects under the LEIF-managed call “Reducing Greenhouse Gas Emissions from Households – Support for the Use of Renewable Energy Sources,” with contributions from several towns and buildings across the country. All buildings receiving financial support were included, with no sampling or selective inclusion process applied.
**Data Source Location**	All relevant data can be found at RETROFIT-LAT's official Github Repositoy: https://github.com/epu-ntua/RETROFIT-LAT ^1^
**Related Research Article**	A. M. Tzortzis *et al.*, “AI4EF: Artificial Intelligence for Energy Efficiency in the building sector,” *SoftwareX*, vol. 30, p. 102172, May 2025, doi: 10.1016/J.SOFTX.2025.102172. ^2^.

## Value of the data

- **Impact of building renovations:** Our data provide valuable geographical information on energy consumption before and after renovations, emissions, and improvements achieved through renovations and upgrades. Researchers can use these features to train models for forecasting the impacts of renovations, enabling scalable and region-specific intervention strategies ^3– 6^
- **Relationship between building design and energy use:** Our data contain multiple characteristics for each building such as floor height, volume, number of floors. Such detail can aid researchers and policymakers understand the correlation between physical factors of a building can influence energy efficiency and consumption. Investors can also benefit from this, using predictive models, in identifying design attributes that maximize energy savings and value ^7, 8^. The ability to leverage advanced models such as transfer learning ^9^ can enhance these predictions and improve their generalizability across different regions. The Artificial Intelligence for Energy Efficiency (AI4EF) ^2^ tool also uses RETROFIT-LAT to improve predictions and suggest optimal design modifications
- **Assessment of Energy Resource Efficiency:** RETROFIT-LAT provides information on different energy sources (e.g., gas, electricity, solid biofuel) and their related *CO*_2 _emission factors. This allows the development of models to compare the efficiency of various energy resources in reducing consumption and emissions. It also aids funding bodies, seeking to allocate resources to the most effective energy strategies and for investors interested in sustainable energy projects ^10^. Machine learning models, as seen in recent works ^11– 13^, can further enhance this analysis by predicting energy efficiency and resource use based on historical data.
- **Renewable Energy and Solar Panel Projects:** RETROFIT-LAT can help stakeholders such as renewable energy companies and government funding programs, to analyse the effectiveness of different project designs, geographic factors influencing solar adoption, and the scalability of solar energy systems in residential and regional contexts ^14, 15^

## Objective

- RETROFIT-LAT’s data were collected by the Latvian Environmental Investment Fund, from several towns and buildings in Latvia. The primary source of data is the Latvian Environmental Investment Fund (LEIF). These datasets provide insights into building energy performance, *CO*_2 _emissions, and renewable energy installations across various projects in Latvia. Through this article, we aim to share the results of these initiatives to support further research, evaluation, and the development of strategies to improve energy efficiency, reduce greenhouse gas emissions, and promote the use of renewable energy in building sectors. For many of the plots generated in the study, an advanced visualization engine for energy visual analytics has been used to illustrate the key results ^6^. This web-based platform, built using React and amCharts, as part of the H2020 MATRYCS project, supports interactive charts, SQL-based queries, and map-based visualizations,. It also includes a role-based access control system to ensure secure and tailored access to building-related data.

## Data description

RETROFIT-LAT is organized into two distinct datasets, each offering unique insights. The first dataset, titled *EF comp*, provides detailed information on building energy efficiency projects, including metadata said buildings (e.g. location, area, volume, and structural features), pre- and post-retrofit energy consumption, carbon dioxide emissions, and technical details of implemented energy-saving measures, The second dataset, named *Sol pan comp* focuses on solar panel installations, capturing data on financial support, electricity consumption from the grid, inverter specifications, solar electricity production, primary energy consumption changes, and *CO*_2_ emissions reductions.

### EF comp dataset overview

The features in RETROFIT-LAT have been organized into distinct categories that provide a comprehensive overview of each building’s energy performance and efficiency measures. These categories include (a) *General Building Information*, (b) *Energy Performance*, (c) *Energy Resource* (d) *CO _2_ Emission and Primary Energy Factors*, (e) *Energy Saving and Efficiency* and (f) *Energy Efficiency Measures*. Each of these categories provides essential data for evaluating the energy performance and environmental impact of buildings before and after interventions.

***General building information***

In Table 1, we display the group of features that offer general details about each building, including its geographic location (region, town/village) and the year it began operation. These features provide essential context for understanding each building’s baseline attributes and forms the basis for analysing and reporting on implemented projects.

**Table 1.** General Building Information.

Feature	Description	Data type
The Date	Date when the project application was submitted and its implementation started.	Text
Region	Planning region where the house is located.	Text
The Town/Village	Town or village where the house is located.	Text
County/City	County or city where the house is located.	Text
Initial year of exploitation	Initial year of exploitation of the building	Integer/Year

This table details the fundamental characteristics of each building in the dataset, focusing on **geographic location** (including planning region, town/village) and the **year the building first began operation**. This information provides essential context for understanding the baseline attributes of the buildings before any retrofitting projects.

**Figure 1.**
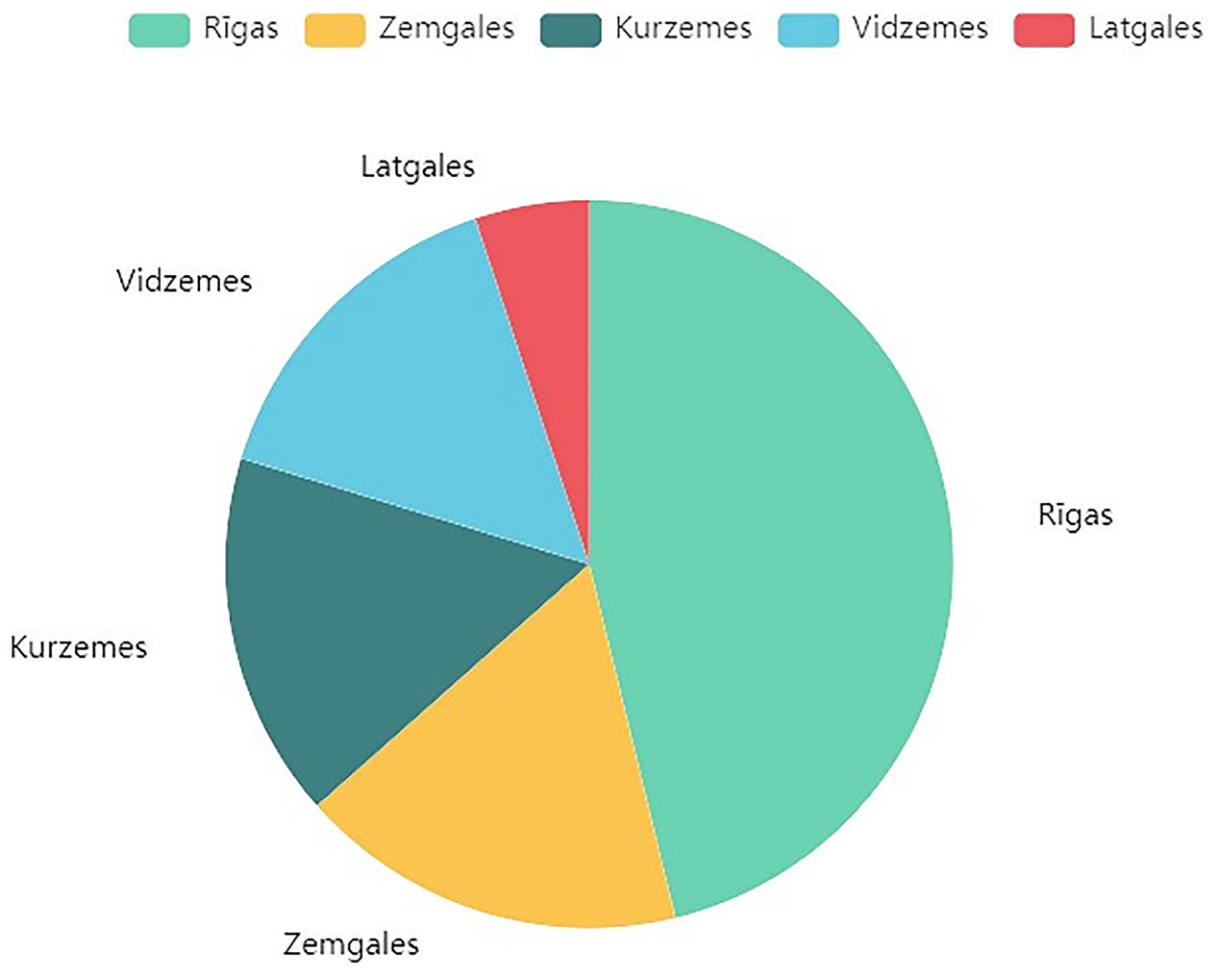
Distribution of data across Latvian regions. Illustrates the geographical spread of the 1010 residential building projects within the five planning regions of Latvia: Riga, Zemgale, Kurzeme, Vidzeme, and Latgale.

The dataset covers five planning regions in Latvia: Riga, Zemgale, Kurzeme, Vidzeme and Latgale, with the distribution of buildings across them shown in Figure 1.

Location is also indicated by the columns. The town/village and County/City, which exhibit a high number of distinct values with 122 and 68 unique entries respectively.

***Building characteristics***

The dataset also encompasses detailed information about the structure of the buildings, offering valuable insights into their impact on energy efficiency and potential for optimization. Table 2 presents the features regarding a building’s physical attributes, thermal performance, and operational parameters. It includes metrics such as Building Total Area, Room Volume, and Reference Area, which are fundamental for energy performance calculations.

**Table 2.** Building Characteristics.

Feature	Description	Unit (measure)
Building Total Area	The total area of the building.	m ^2^
Room Volume	The total volume of rooms in the building.	m ^3^
Average Floor Height	The average height of each floor.	m
Reference Area	The reference area used in energy performance calculations.	m ^2^
Above-Ground Floors	The number of floors above ground level in the building.	Floor number
Underground Floor	Indicates whether the building has underground floors.	Yes/No
Mansard	Indicates if the building has a mansard roof.	Yes/No
Roof Floor	Indicates if the building has a roof floor.	Yes/No
Area of the External Surface	The total area of the external surface of the building.	m ^2^
Average Heat Transfer Coefficient	The average heat transfer coefficient, which indicates the insulation performance.	W/(m ^2^K)
Average Heat Transfer Coefficient _1_	An alternative value of the heat transfer coefficient for different parts of the building.	W/(m ^2^K)
Building Calculated Heat Loss Coefficient	The calculated value for the heat loss coefficient of the building.	W/(m ^2^K)
Building Allowable Heat Loss Coefficient	The allowable value for the building’s heat loss coefficient based on regulations.	W/(m ^2^K)
Indoor Temperature Heating	The indoor temperature set for heating.	°C
Indoor Temperature for Cooling	The indoor temperature set for cooling.	°C
Air Exchange Rate	The rate at which air is exchanged in the building.	*h*−1
Ventilation Heat Loss Coefficient	The coefficient that quantifies the heat loss due to ventilation.	W/(m ^2^K)

This table presents a comprehensive overview of the **physical attributes, thermal performance metrics, and operational parameters** of the buildings in the study. Key features include **building size** (total area, room volume, reference area), **structural details** (average floor height, number of above-ground/underground floors, presence of a mansard or roof floor, external surface area), **thermal insulation properties** (average heat transfer coefficients, calculated and allowable heat loss coefficients), and **operational settings** (indoor temperatures for heating and cooling, air exchange rate, ventilation heat loss coefficient).

Structural details related to the buildings’ flooring (e.g average floor height, the number of above-ground/underground floors) provide important insights into the building’s design. As a general overview, 66% of the buildings have two above-ground floors. While only 7 buildings include three floors. The remaining buildings consist of a single floor and an underground floor is present in 42% of the properties. A roof floor exists in approximately 14% of the houses and 24% are constructed with a mansard roof, indicating varied architectural designs that contribute to the dataset’s diversity.

Thermal performance is evaluated through metrics (e.g average heat transfer/loss coefficients etc), which reflect the building’s insulation and compliance with energy standards. Operational settings (e.g indoor temperatures for heating and cooling, Air Exchange Rate etc) are critical parameters for assessing energy consumption and ensuring thermal comfort.

***Energy performance***

**Figure 2.**
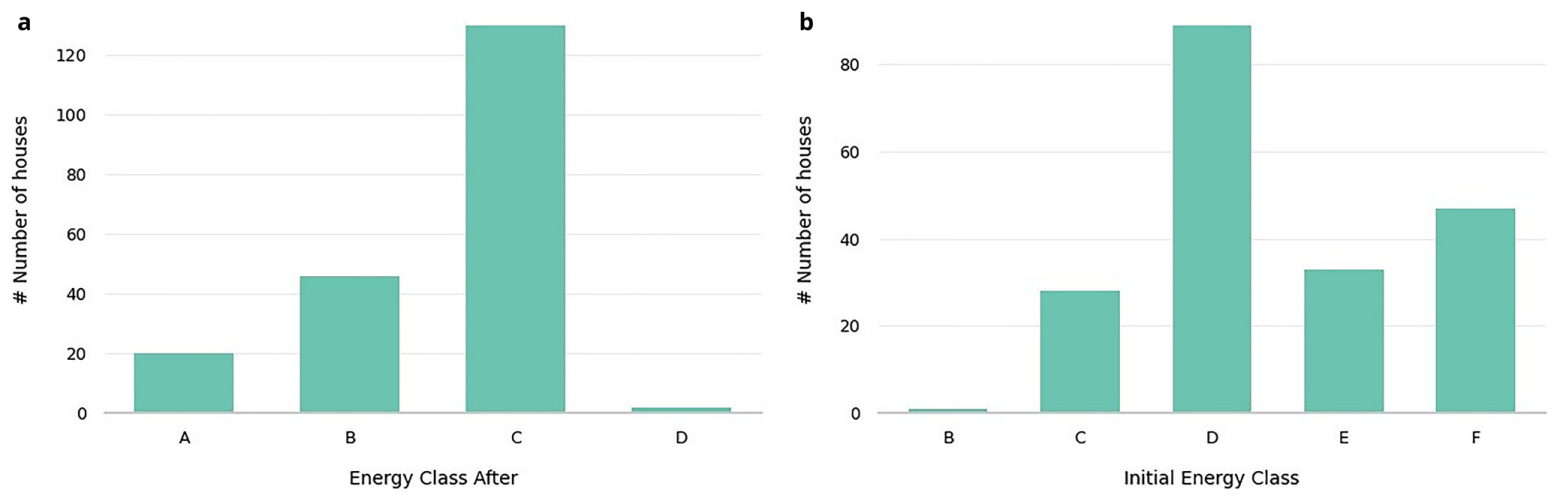
Initial energy class and Energy class after distributions. Showcases the shift in the energy efficiency of the buildings before and after retrofitting interventions.

Energy performance features, such as Energy Class (Initial Energy Class, Energy Class After), which reflect the building’s energy efficiency before and after improvements, are illustrated in Table 3. In Figure 2, we present the distribution of buildings’ energy classes before and after renovations, highlighting the impact of these interventions on energy efficiency. Initially most buildings belonged to classes D, E, and F. After the retrofitting, all buildings were upgraded to classes A (10%), B (22%), C (68%).

**Table 3.** Energy Performance.

Feature	Description	Unit (Measure)
Energy Consumption Before	The total energy consumption of the building before energy-saving interventions.	*kWh/m* ^2^
Initial Energy Class	The energy class of the building before	Energy Class (e.g. A,B,C)
Heat Energy Consumption Before	The total heat energy consumption of the building before the renovation.	*kWh/m* ^2^
Carbon Dioxide Emissions Before	The carbon dioxide emissions from the building’s energy consumption before renovation.	kg *C*O _2_ */m* ^2^ per year
Energy Consumption After	The total energy consumption of the building after energy-saving interventions.	*kWh/m* ^2^
Energy Class After	The energy class of the building after	Energy Class (e.g. A,B,C)
Heat Energy Consumption After	The total heat energy consumption of the building after renovation.	*kWh/m* ^2^
Consumption for Hot Water After	The energy consumption for hot water after energy-saving interventions.	*kWh/m* ^2^
Consumption for Mechanical Ventilation After	The energy consumption for mechanical ventilation after interventions.	*kWh/m* ^2^
Consumption for Lighting After	The energy consumption for lighting after renovation.	*kWh/m* ^2^
Consumption for Cooling After	The energy consumption for cooling after renovation.	*kWh/m* ^2^
Primary Non-Renewable Energy	The amount of non-renewable energy used after renovation.	*kWh/m* ^2^
Primary Total Energy Consumption	The total amount of energy consumed after renovations, considering all sources.	*kWh/m* ^2^
Almost Zero Energy Building	Indicates if the building meets almost zero energy standards after renovation.	Yes/No
Carbon Dioxide Emission Tons After	The total carbon dioxide emissions after energy- saving interventions.	tons CO _2_ per year
Carbon Dioxide Emission After	The carbon dioxide emissions after renovations.	kg *C*O _2_ */m* ^2^ per year

This table outlines the **energy consumption and environmental impact** of the buildings, both **before and after** the implementation of energy-saving interventions. It includes the **initial and final energy classes**, **total and heat energy consumption**, **consumption for specific uses after renovation** (hot water, mechanical ventilation, lighting, cooling), **primary energy consumption** (non-renewable and total), and **carbon dioxide emissions**. It also indicates whether a building meets **almost zero energy building standards** after renovation.

**Figure 3.**
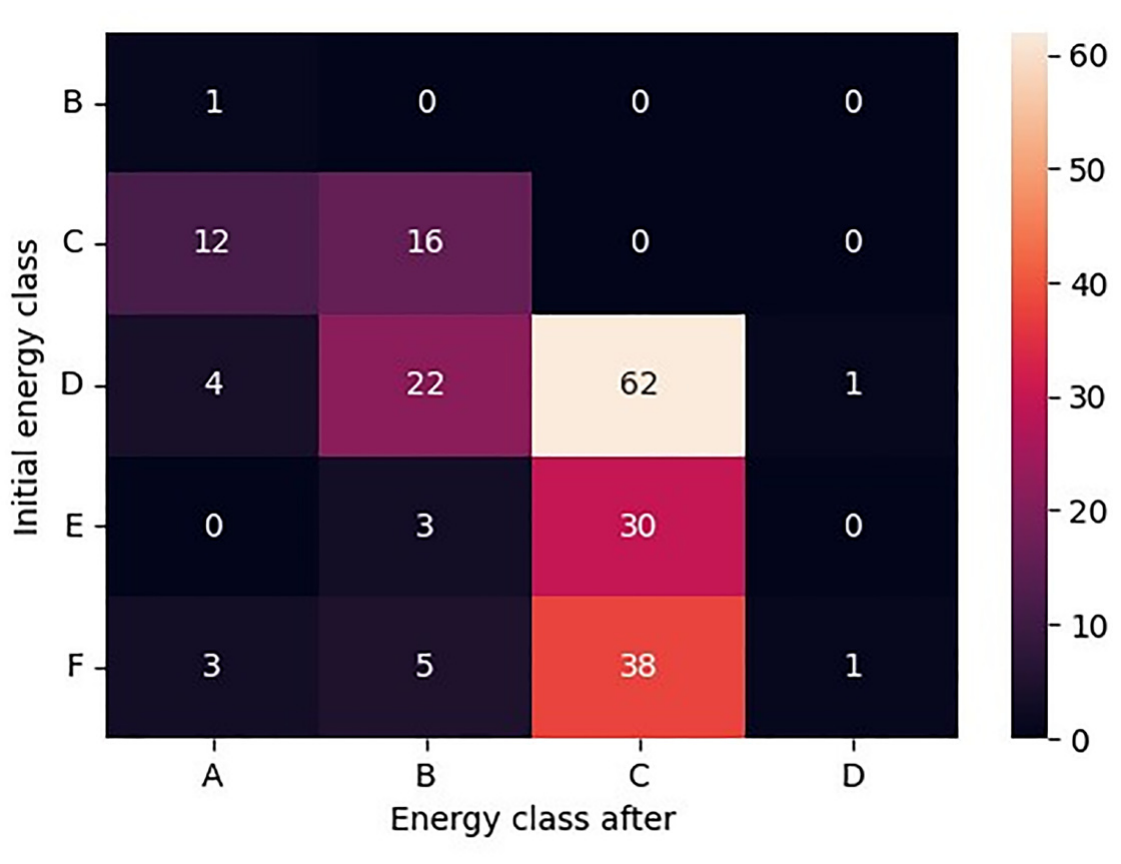
Heatmap of Initial energy class and Energy class after combinations. A detailed view of the transitions between the initial and final energy classes for the retrofitted buildings, illustrating the specific patterns of energy class progression within the dataset.

Specifically, the heatmap in Figure 3 illustrates the transitions between initial and final energy classes in detail, highlighting the specific patterns of energy class progression within the dataset.

**Figure 4.**
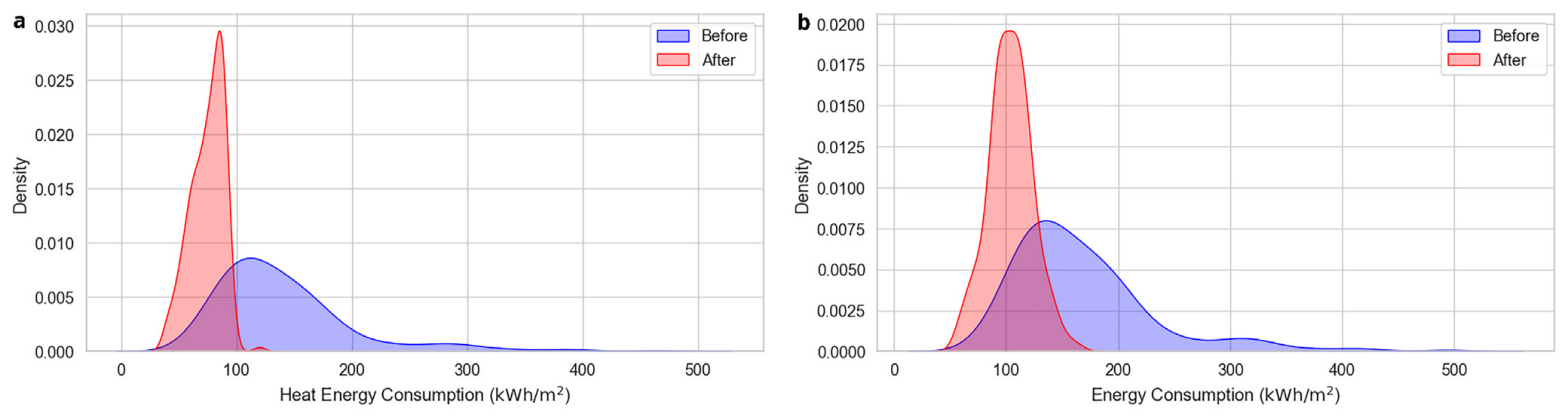
Total and heat energy consumptions before and after the renovation. Visualizes ( **a**) the distribution of total energy consumption before and after the implementation of energy efficiency measures ( **b**) a similar pattern for heat energy consumption, which represents the largest share of total energy demand.

The Energy Consumption (e.g., Energy Consumption Before, Energy Consumption After) measures the total energy used by the building. Figure 4a illustrates the distribution of total energy consumption both before and after the implementation of energy efficiency measures, capturing a significant reduction in their values. As shown in Figure 4b, a similar pattern is observed in the case of energy consumption for heating, which accounts for the largest share of total energy demand.

This group of features also includes specific categories regarding energy consumption (e.g. consumption for mechanical ventilation/lighting/cooling) highlighting the changes resulting from energy-saving interventions in each system. The Primary Energy (e.g., Primary Non-Renewable Energy, Primary Total Energy Consumption) measures the primary energy usage from both renewable and non-renewable sources after renovation, with Primary Non-Renewable Energy focusing on non-renewable resources.

**Figure 5.**
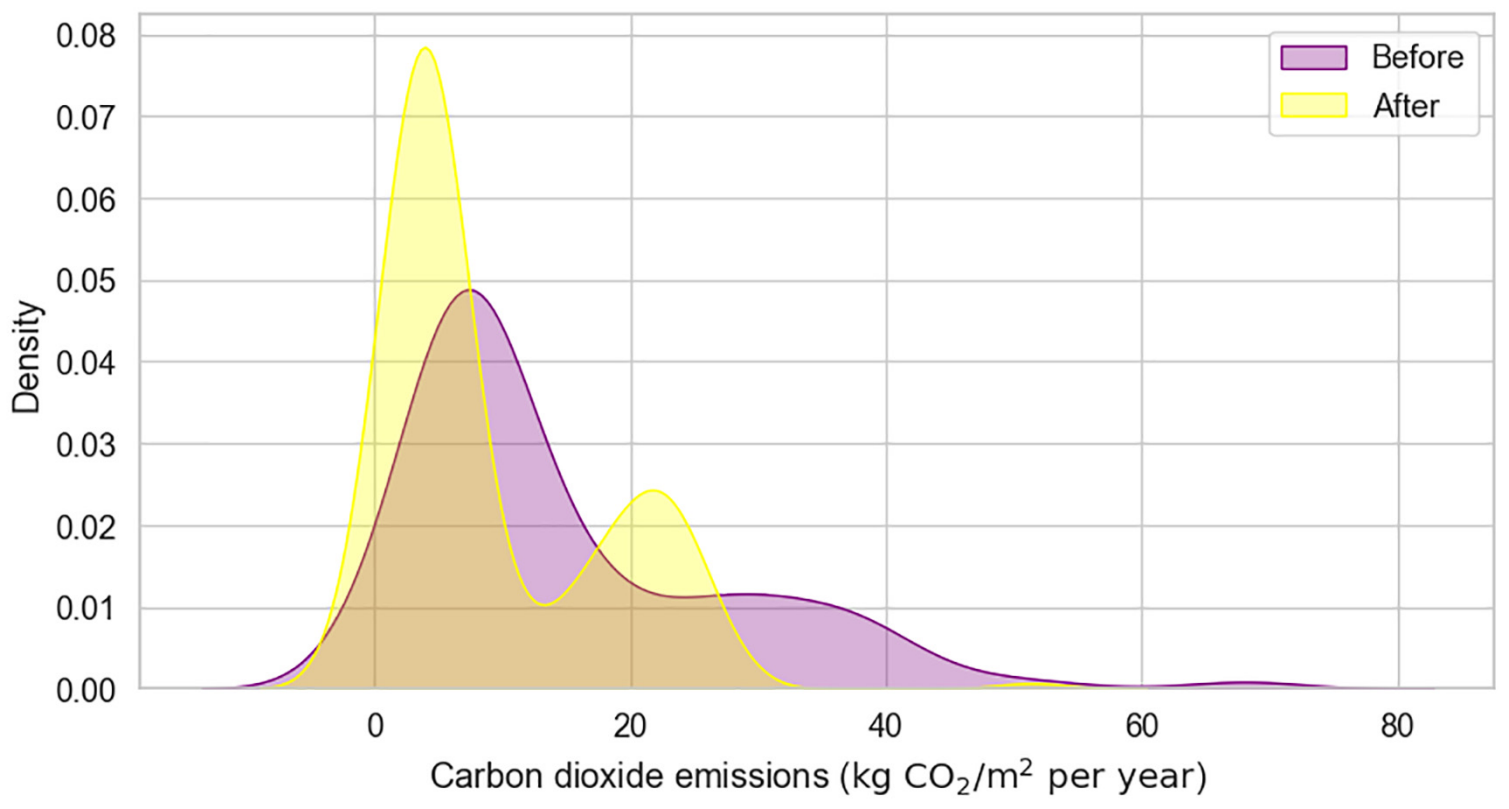
KDE plot of Carbon dioxide emissions before and after the renovation. Visualizes the distributions of CO _2_ emissions associated with the buildings' energy consumption.

Carbon Dioxide Emissions (e.g. Carbon Dioxide Emissions Before, Carbon Dioxide Emissions After) track the amount of *CO*_2_ emissions associated with the building’s energy consumption before and after renovations. Figure 5 visualizes the corresponding distributions, emphasizing the environmental benefits of the retrofitting actions. Lastly, the Almost Zero Energy Building feature indicates whether the building meets almost zero energy standards after renovation.

***Energy saving and efficiency***

The Energy Saving and Efficiency category, illustrated in Table 4, focuses on the impact of energy-saving measures implemented in the building. It includes features such as Heat Saving for Heating, which measures the reduction in heat energy consumption for heating purposes after renovations. Total Energy Consumption Saving tracks the overall energy savings achieved across all systems and interventions, providing a comprehensive view of the building’s energy efficiency improvements. Additionally, Saving of Heat Energy specifically quantifies the amount of heat energy conserved through various energy-saving measures. These features collectively help assess the effectiveness of renovations in reducing energy usage, improving efficiency, and lowering operational costs.

**Table 4.** Energy Saving and Efficiency.

Feature	Description	Unit (Measure)
Heat Saving for Heating	The amount of heat energy saved for heating after interventions.	*kWh/m* ^2^
Total Energy Consumption Saving	The total energy saved after renovations.	*kWh/m* ^2^
Saving of Heat Energy	The total amount of heat energy saved through energy-saving measures.	%

This table focuses specifically on the **quantifiable impact of the energy efficiency measures** implemented in the buildings. It presents metrics such as **heat energy saved for heating**, **total energy consumption saving**, and the **percentage saving of heat energy** achieved through the retrofitting actions.

***Energy resource use***

Features describing energy resource uses (e.g heating/hot water, ventilation etc) provide insight into the primary and secondary energy sources used to power key building systems. These features help in understanding the overall energy mix and efficiency of the building, capturing different resources utilized. Table 5 presents these features in detail, outlining the proportions of energy sources such as electricity, natural gas, or renewable options, offering a clear perspective on resource allocation. Note that in many buildings distinct energy resources are not allocated for specific systems such as ventilation. Similarly, the presence of a secondary energy resource for heating, hot water, or cooling is not applicable in certain cases and, therefore, the corresponding features are assigned the value ’-’. This data is crucial for evaluating sustainability and optimizing energy consumption across multiple buildings.

**Table 5.** Energy Resource Use.

Feature	Description	Data type
Energy Resource for Heating 1	The primary energy resource used for heating (e.g., gas, electricity).	Text
Energy Resource for Heating 2	The secondary energy resource used for heating.	Text
Energy Resource for Hot Water 1	The primary energy resource used for hot water.	Text
Energy Resource for Hot Water 2	The secondary energy resource used for hot water.	Text
Energy Resource for Ventilation	The primary energy resource used for the ventilation system.	Text
Energy Resource for Cooling 1	The primary energy resource used for cooling the building.	Text
Energy Resource for Cooling 2	The secondary energy resource used for cooling.	Text

This table provides insights into the **primary and secondary energy sources** utilised to power key building systems, including **heating**, **hot water**, **ventilation**, and **cooling**. It lists the specific energy resources (e.g., gas, electricity, renewable options) used for each system. The use of '-' indicates instances where a distinct energy resource is not allocated for a specific system or where a secondary energy resource is not applicable.

***CO _2_ emission and primary energy factors***

Features regarding *CO*_2_ emission factors (e.g heating, hot water, ventilation etc.) indicate the amount of carbon dioxide emissions associated with the energy consumed by building energy systems. These factors, depicted in Table 6 are based on the specific energy sources used in each system, providing a measure of their environmental impact. Similarly, non-renewable/renewable energy factors for said energy systems capture the non-renewable/renewable energy consumed by these systems. The total factors are derived as the sum of the corresponding non-renewable and renewable factors. This data also assist in the evaluation of a building’s sustainability, supporting decisions towards reducing emissions and improving energy efficiency.

**Table 6.** CO _2_ Emission and Primary Energy Factors (X refers to HVAC systems: heating 1, heating 2, hot water 1, hot water 2, ventilation, cooling 1, cooling 2).

Feature	Description	Data type
CO _2_ emission factor for *X*	CO _2_ emission factor for *X* based on the energy source used.	Integer
Non-renewable factor for *X*	Primary energy factor for the part of non-renewable energy resources for *X*.	Integer
Renewable factor for *X*	Primary energy factor for the part of renewable energy resources for *X*.	Integer
Total factor for *X*	Total primary energy factor for *X* based on the energy source used.	Integer

This table details the **carbon dioxide emission factors** associated with the energy consumed by different HVAC (Heating, Ventilation, and Air Conditioning) systems, based on their specific energy sources. It also presents the **non-renewable and renewable primary energy factors** for these systems, as well as the **total primary energy factor**, offering a measure of the environmental impact and sustainability of each system.

***Energy efficiency measures***

Finally, we have retrofitting actions (e.g Carrying out Construction Works, Reconstruction of Engineering Systems etc) that represent different energy efficiency measures in building renovation and upgrades. These measures focus on improving the overall energy performance of the building. For instance, *Carrying out Construction Works* refers to the work done on enclosing structures, while *Reconstruction of Engineering Systems* involves upgrading systems like ventilation or recuperation to boost energy efficiency. The *Water Heating System* refers to the installation of a new system to improve hot water energy use, and *Heat Installation* focuses on ensuring that heating is produced using renewable energy sources. Table 7 illustrates these measures in detail, highlighting their significance as crucial steps in enhancing a building’s sustainability and energy efficiency.

**Table 7.** Energy efficiency measures.

Feature	Description	Data type
Carrying out construction works	Carrying out construction works in the enclosing structures	Boolean
Reconstruction of engineering systems	Reconstruction of engineering systems (ventilation, recuperation) to increase the energy efficiency of the house	Boolean
Water heating system	Installation of a new water heating system	Boolean
Heat installation	Installation of heat installations to ensure the production of heat from renewable energy sources	Boolean

This table describes the various **retrofitting actions** undertaken to improve the energy efficiency of the buildings. These measures include **carrying out construction works on enclosing structures**, **reconstruction of engineering systems** (ventilation, recuperation), **installation of new water heating systems**, and **installation of heat installations** to utilize renewable energy sources for heating.

**Figure 6.**
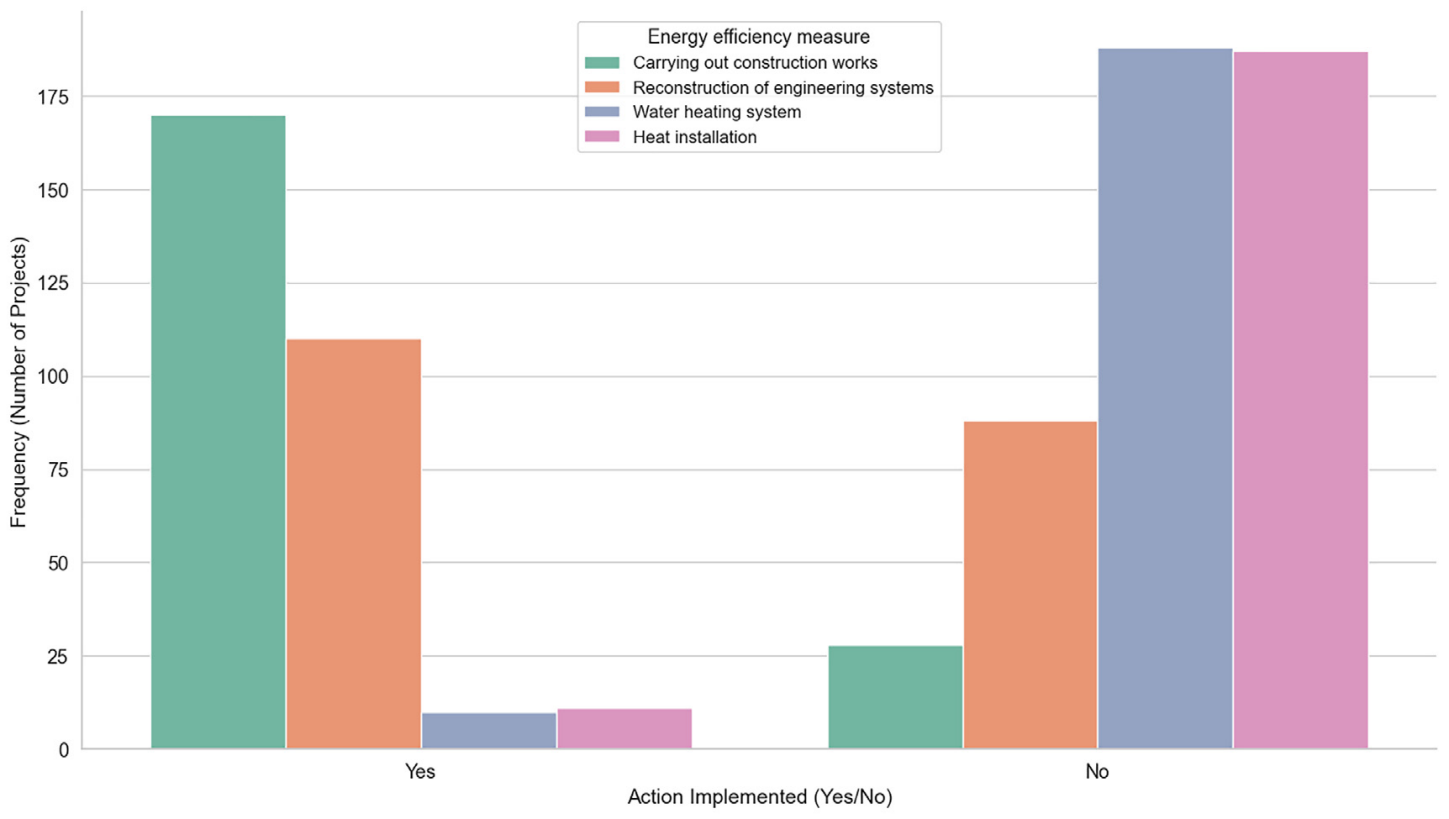
Clustered Bar Plot for Energy efficiency measures implementation. Comparison between the number of buildings that implemented each of the energy efficiency measures (Carrying out Construction Works, Reconstruction of Engineering Systems, Water heating system, Heat installation) to those that did not, providing an overview of the adoption rates for these retrofitting actions.

Figure 6 visualizes the number of buildings that implemented each energy efficiency measure compared to those that did not, providing an overview of the adoption rates for these retrofitting actions.

### Sol pan comp dataset overview

**Figure 7.**
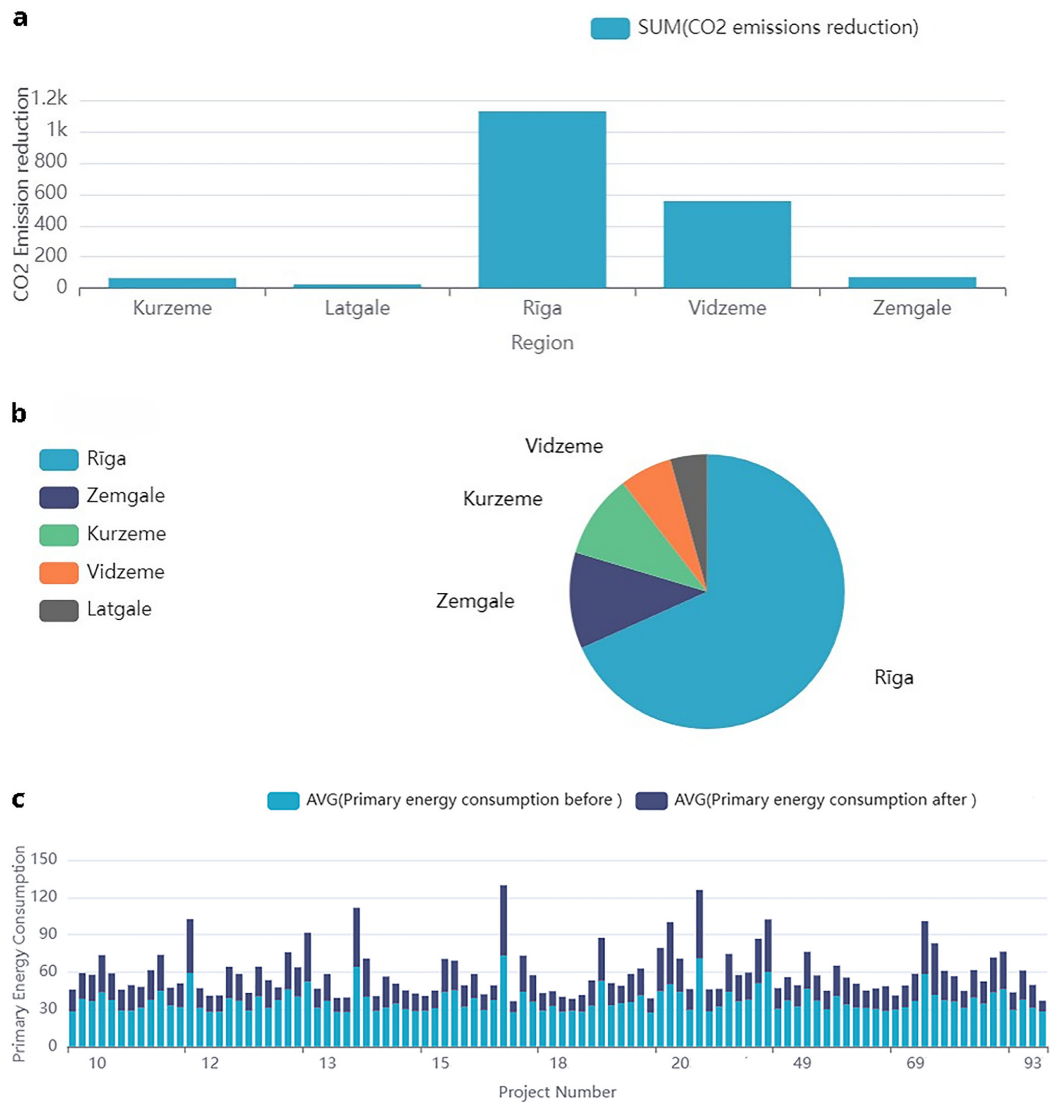
Solpancomp: Analysis of energy efficiency measures, including emissions reduction, data distribution, and energy consumption metrics. This figure presents various analyses related to the solar panel installation dataset: ( **a**) shows CO _2_ emissions reduction by region, ( **b**) illustrates the data distribution by region for solar panel projects, and ( **c**) displays the primary energy consumption before and after implementation of solar panel systems.

The dataset contains detailed information regarding solar panel projects, funded through public support in Latvia, that focus on energy efficiency and sustainability metrics. Key features include the region attribute which categorizes projects by Latvia’s five planning regions, enabling regional analysis. Quantitative data include pre- and post-project electricity consumption (MWh/year), and primary energy consumption (kW), allowing assessment of energy-saving actions. The dataset also records solar panel electricity production (MWh/year), alongside *CO*_2 _emissions reduction (t CO _2_ eq/year) as an environmental impact metric ( Figure 7). Table 8 provides a detailed overview of these features, including their descriptions and units of measure. This comprehensive dataset is valuable for analysing the impact of solar energy projects on energy efficiency and greenhouse gas reduction.

**Table 8.** Feature Descriptions for Solar Panel comp dataset.

Feature	Description	Unit (measure)
The date	The year when the project application was submitted and its implementation started.	Text
Region	The planning regions where the house is located.	Text
Electricity consumption from the grid	Electricity consumption from the grid before the project.	MWh/year
Primary energy consumption before	Primary energy consumption before the installation of the solar panel system during the project.	kW
Current inverter set power	Current inverter set power - inverter power that was already installed before the project.	kW
Inverter set power in project	Inverter set power in project - in addition to the existing inverter.	kW
Electricity produced by solar panels	The amount of electricity produced by the solar panels, which are installed in the project.	MWh/year
Primary energy consumption after	Primary energy consumption after installing the solar panel system.	MWh/year
Reduction of primary energy	Reduction of primary energy.	MWh/year
CO _2_ emissions reduction	CO _2_ emissions reduction.	tons CO _2_ eq/year

This table provides a detailed overview of the features included in the solar panel installation dataset. It describes attributes such as the **project submission year**, **planning region**, **electricity consumption from the grid** (before and after the project), **primary energy consumption** (before and after installation), **current and project-related inverter power**, **electricity produced by solar panels**, **reduction of primary energy**, and **CO2 emissions reduction**.

## Experimental design, materials and methods

In the data preprocessing stage, several essential steps were undertaken to ensure data consistency across the dataset. Basic edits were applied to eliminate inconsistencies and harmonize formatting (e.g. different value representations, translation of entries remaining in Latvian etc.). Energy classes were validated and corrected to align with the Latvian energy efficiency system ^16^. As regards the null values, the value ’-’ was retained to indicate the non-existence of an energy system (and energy factor respectively), while missing values were preserved to allow users to handle them as needed.

## Limitations

RETROFIT-LAT exhibit some common limitations. Both of its datasets are of limited size, recording combined data from only 1010 buildings. Another key issue is data scarcity, where specific features such as Latvian region, energy class, and energy efficiency measures, are under-represented. This imbalance is illustrated more notably in Figure 1, Figure 2 and Figure 6 respectively.

## Ethics statement

It is clearly stated that the work carried out for the needs of the data collection and presentation does not involve human subjects, animal experiments, or any data collected from social media platforms. It should be further clarified that no personal data are included in RETROFIT-LAT concerning the participant buildings’ stakeholders and that the platform(s)’ data redistribution policies are fully complied with it.

## Data Availability

Due to ethical and privacy considerations, the dataset cannot be fully shared in its original form. The study protocol and data handling procedures were reviewed by LEIF, which required that public data release be restricted. To minimize privacy risks, only minimal modifications were made. Columns containing personally identifiable information (PII), including *Project number*, *Granted support, Home address* and *Energy audit number* were entirely removed. Location-related data in the *The town/village* and *County/City* columns was pseudo-anonymized by replacing unique values with sequential labels (e.g town1, town2, and county1 county2 respectively) Furthermore, the *The date* column was adjusted to display only the year, omitting specific day and month details. These steps are important to ensure compliance with data protection standards, homeowner’s individual privacy, and maintain the dataset’s utility for meaningful analysis. Access to the full dataset may be granted upon request, subject to approval by LEIF, and with the signing of a data use agreement. Researchers interested in accessing the full dataset can contact the authors for more details. A limited version of the dataset is publicly available under an open **CC-BY 4.0** license at RETROFIT-LAT’s official Github repository ^1^ and is assigned the **Persistent Identifier (DOI)**: 10.5281/zenodo.14697230. This version excludes sensitive information and includes only the anonymized data, which still allows for meaningful analysis. Zenodo: RETROFIT-LAT: A comprehensive dataset for energy efficiency investments in Latvia, DOI: https://doi.org/10.5281/zenodo.15316421 () The project contains a limited version of the dataset is publicly available. 1. epu-ntua/RETROFIT-LAT-1.1.2.zip Data are available under the terms of the Creative Commons Attribution 4.0 International license (CC-BY 4.0).
