# Supplementary material for: RETROFIT-LAT: A comprehensive dataset for energy efficiency investments in Latvia
Source: Open Res Eur. 2025 Jul 31;5:134. Originally published 2025 May 16. [Version 2] doi: 10.12688/openreseurope.20042.2 (PMC12438949; doi:10.12688/openreseurope.20042.2)
Supplement: Peer review history

# Reviewer report: Reviewer response for version 2

**DOI:** 10.21956/openreseurope.22793.r58937

**Published:** 2025-09-15

**Authors:** Jiapeng Dai

**Affiliations:** 1 University of Wollongong Malaysia, Shah Alam, Malaysia

This revised data note is useful, clearly structured, and much closer to indexing. The two linked datasets (retrofits and PV projects) are described with enough context to be actionable, the figures effectively show before/after shifts in consumption and emissions, and the ethics/data-sharing sections are transparent. What remains are small but important fixes to tighten technical consistency, documentation, and presentation so downstream users can reproduce and trust the resource. I recommend **minor revision** focused on the points below. 

 First, resolve unit and scale inconsistencies in the PV table and text so variables are unambiguous. In Table 8 the “primary energy consumption before” appears in kW while the “after” value is in MWh/year; both should use the same energy unit (likely MWh/year), and the text on page 10–12 should match the table. Also check that “current inverter set power” and “inverter set power in project” are consistently named and unit-labeled. 

 Second, fix DOI and repository references so there is a single, canonical citation and version tag. The data availability section mentions two different Zenodo DOIs and a GitHub zip name; please standardize on one DOI (latest deposition) and one release tag, and mirror that in the Specifications table and the abstract.

 Third, polish data types, units, and dictionary entries in the HVAC factors table. Table 6 lists CO₂ and primary energy “factors” as “Integer,” but in practice they will often be decimals with explicit units (e.g., kg CO₂/kWh; dimensionless primary energy factors). Update the data types to numeric (float), add units, and clarify whether factors are per-kWh of final energy by system. This will prevent misinterpretation by modelers.

 Fourth, tighten the methodological description for preprocessing so readers can replicate the public release exactly. You already note translation, validation of energy classes, and pseudo-anonymization; please add (i) the precise cleaning/standardization steps applied (normalization, outlier handling, winsorizing, if any), (ii) the rule for preserving “-” versus NA, and (iii) a short code snippet or script names in the repo that reproduce the published CSVs from raw LEIF extracts. A compact, machine-readable data dictionary (CSV/README) summarizing variable names, units, allowed values, and missing-data conventions would be very helpful.

 Fifth, ensure figures and captions align with the claims in the text and carry complete axis labels/units. For example, the bar and heatmap panels for energy classes (pages 6–7) should explicitly state counts or percentages and match the statement that upgrades concentrate in classes A–C. In Figure 7c, harmonize the “AVG” labeling and units on the y-axis so the before/after comparison is immediately clear.

 Sixth, standardize regional and categorical labels across text, tables, and figures. The pie chart on page 4 uses regional names with inconsistent pluralization; use one convention (Riga, Zemgale, Kurzeme, Vidzeme, Latgale) everywhere. Similarly, keep capitalization and hyphenation of variable names (e.g., “Energy Class After,” “Primary energy consumption after”) consistent across tables and the CSV headers.

 Seventh, clarify sample coverage and selection in one place to pre-empt confusion. The revision states that the dataset covers all completed projects under a specific LEIF call and that no sampling was applied; add the call’s exact dates, eligibility criteria, and a one-line inclusion flow (eligible → realized → included in public release) so users understand population vs. sample. If some variables are sparse (as noted in the Limitations), consider a small completeness table by field.

 Addressing these light edits will make the dataset easier to cite, parse, and reuse without changing its substance. With those in place, I support indexing.

Are sufficient details of methods and materials provided to allow replication by others?

Yes

Is the rationale for creating the dataset(s) clearly described?

Yes

Are the datasets clearly presented in a useable and accessible format?

Yes

Are the protocols appropriate and is the work technically sound?

Yes

Reviewer Expertise:

Energy economics and environmental policy

I confirm that I have read this submission and believe that I have an appropriate level of expertise to confirm that it is of an acceptable scientific standard, however I have significant reservations, as outlined above.

# Reviewer report: Reviewer response for version 2

**DOI:** 10.21956/openreseurope.22793.r57548

**Published:** 2025-09-15

**Authors:** Darlington Eze Ekechukwu

**Affiliations:** 1 University of East London, London, England, UK

Manuscript is very detailed and well presented, showing scholarly relevant dataset to can be used to advance the energy efficiency drive within the power sector not just in Latvia but in many other regions of the world.

Are sufficient details of methods and materials provided to allow replication by others?

Yes

Is the rationale for creating the dataset(s) clearly described?

Yes

Are the datasets clearly presented in a useable and accessible format?

Yes

Are the protocols appropriate and is the work technically sound?

Yes

Reviewer Expertise:

NA

I confirm that I have read this submission and believe that I have an appropriate level of expertise to confirm that it is of an acceptable scientific standard.

# Reviewer report: Reviewer response for version 1

**DOI:** 10.21956/openreseurope.21678.r55909

**Published:** 2025-07-03

**Authors:** Muhammad Shafique

**Affiliations:** 1 Brunel University London, Middlesex, UK

OVERALL REVIEW RESULT

 This is a well-structured and written article presents RETROFIT-LAT: a collection of data from 1010 residential building projects, funded by the Republic of Latvia’s Environmental Investment Fund (LEIF). However, as the main drawback, it isn't easy to recognize the importance of the research results. Reading the conclusions, the study has not contributed significantly to new findings that are not already available in the existing literature, and the overall outcome sounds trivial. Also, I believe it is very important to highlight content on the importance of incorporating methods. 

 I hope that this feedback will be helpful as you look to strengthen your research efforts.

 MORE SPECIFIC COMMENTARY

 The paper aims to provide the first dataset analyses the energy performance and sustainability of buildings before and after retrofitting actions, including their energy consumption, *CO*_2_ emissions, and energy classes. It spans projects implemented from 1870 to 2022, covering various building types and regions, with data on their energy use, heat loss coefficients, and both renewable and non-renewable energy contributions.

 The structure of this paper is suitable.

 The English language used in the manuscript is good enough to convey the idea of the paper without critical mistakes in grammar, word selection, and typing. Still, a second English proofread is suggested.

 Title - It is reasonable.

 Abstract—It still can improve. The abstract is heavily built on outcomes and does not provide information on aims. Instead, I recommend structuring a more detailed aim that the outcome can follow.

 Keywords - They are reasonable.

 It is important to clearly give detail information on methods selection for methodology section. 

 While the work appears technically solid and well-organized, with relevant features and structured categories, the data collection and processing protocols are not described in sufficient technical detail. The article lacks the following:

  ○ Clarification on whether data were normalized or standardized.

 ○ No mention of data cleaning, anonymization, or aggregation methods.

 ○ Uncertainty regarding the temporal resolution of data.

  ○ The tool or platform used for data processing and visual analytics is mentioned vaguely but not named or described.

  ○ As energy consumption, saving and CO2 emission etc depends on many factor such as weather conditions, sunlight, ventilation, advance technologies and others, does authors consider these factors for their analysis? 

  ○ Figure 7. Solpancomp: Analysis of energy efficiency measures, including emissions reduction, data distribution, and energy consumption metrics. This figure presents various analyses related to the solar panel installation dataset: (a) shows CO2 emissions reduction by region, this CO2 emissions  are per year or per life cycle?

Are sufficient details of methods and materials provided to allow replication by others?

Partly

Is the rationale for creating the dataset(s) clearly described?

Yes

Are the datasets clearly presented in a useable and accessible format?

Partly

Are the protocols appropriate and is the work technically sound?

Partly

Reviewer Expertise:

Sustainability, Life-cycle Energy and Environmental Assessment (LCA), Circular Economy, Sustainable Construction

I confirm that I have read this submission and believe that I have an appropriate level of expertise to confirm that it is of an acceptable scientific standard, however I have significant reservations, as outlined above.

# Reviewer report: Reviewer response for version 1

**DOI:** 10.21956/openreseurope.21678.r54658

**Published:** 2025-06-13

**Authors:** Muhyiddine Jradi

**Affiliations:** 1 University of Southern Denmark, Odense, Denmark

The article presents RETROFIT-LAT, a dataset composed of detailed information from 1010 residential building retrofit projects funded by the Latvian Environmental Investment Fund (LEIF). It consists of two datasets:

1. EF comp: Focuses on general and technical data about buildings, energy consumption, emissions, and retrofitting measures.
2. Sol pan comp: Focuses on buildings with photovoltaic (PV) systems, capturing pre/post electricity usage, system specifications, and CO₂ emissions.

The data spans from 1870 to 2022, covers all major Latvian regions, and provides both geographic and technical diversity. The authors highlight the dataset’s utility for researchers, policymakers, and investors in energy efficiency, machine learning applications, and renewable energy deployment analysis.

 The RETROFIT-LAT dataset offers high potential value for the research and policymaking communities in energy retrofitting and sustainability. The conceptual rationale is solid, and the dataset appears to be comprehensive and relevant. However, the article currently lacks methodological transparency and technical reproducibility, which are essential for scientific rigor.

 The article clearly establishes the importance of building renovation in improving energy efficiency and reducing emissions. It aligns RETROFIT-LAT with national and EU-level sustainability goals, underlining its relevance for a wide range of stakeholders such as researchers, government agencies, and energy investors. The potential use cases in ML model development and energy policy design are convincingly outlined in both the abstract and objectives sections.

 While the work appears technically solid and well-organized, with relevant features and structured categories, the data collection and processing protocols are not described in sufficient technical detail. The article lacks the following:

- Information on quality control procedures, especially how missing or inconsistent entries were handled.
- Clarification on whether data were normalized or standardized.
- No mention of data cleaning, anonymization, or aggregation methods.
- Uncertainty regarding the temporal resolution of data.

Additionally, the article presents detailed descriptions of variables and features within the datasets, along with insightful visualizations. However, key methodological aspects required for full replication are missing:

- The process for data acquisition from LEIF is not described in detail.
- It is unclear how buildings were selected for inclusion in the dataset, was it random, complete coverage, or conditional based on data availability?
- The tool or platform used for data processing and visual analytics is mentioned vaguely but not named or described.

Are sufficient details of methods and materials provided to allow replication by others?

Partly

Is the rationale for creating the dataset(s) clearly described?

Yes

Are the datasets clearly presented in a useable and accessible format?

Yes

Are the protocols appropriate and is the work technically sound?

Partly

Reviewer Expertise:

Building energy services, smart buildings, digital solutions, HVAC systems, sustainable energy technologies

I confirm that I have read this submission and believe that I have an appropriate level of expertise to state that I do not consider it to be of an acceptable scientific standard, for reasons outlined above.
